# Concurrence of dermatomyositis and psoriasis: a case report and literature review

**DOI:** 10.3389/fimmu.2024.1345646

**Published:** 2024-01-29

**Authors:** Dan Chu, Wei Yang, Jun Niu

**Affiliations:** Department of Dermatology, General Hospital of Northern Theater Command, Shenyang, China

**Keywords:** psoriasis, dermatomyositis, pathogenesis, corticosteroid, case report

## Abstract

Dermatomyositis (DM) is a type of inflammatory myopathy with unknown causes. It is characterized by distinct skin lesions, weakness in the muscles close to the body, and the potential to affect multiple organs. Additionally, it may be associated with the presence of malignancies. The development of DM is influenced by genetic susceptibility, autoimmune response, and various external factors like cancer, drugs, and infectious agents. Psoriasis is a chronic, recurring, inflammatory, and systemic condition. Scaly erythema or plaque is the typical skin manifestation. The etiology of psoriasis involves genetic, immune, environmental and other factors. It is uncommon for a patient to have both of these diseases simultaneously, although individuals with DM may occasionally exhibit symptoms similar to those of psoriasis. Our patient was diagnosed with psoriasis in his 50s because of scalp squamous plaques, but he did not receive standard treatment. Ten years later, he developed symptoms of muscle pain and limb weakness. He was diagnosed with psoriasis complicated with dermatomyositis in our department and received corresponding treatment. Moreover, we reviewed the relevant literature to evaluate similarities and differences in clinical manifestation and treatment to other cases.

## 1 Introduction

Dermatomyositis (DM), an idiopathic inflammatory condition involving muscles and skin, is characterized by varying degrees of skin, muscle, and visceral organ involvement. The laboratory assay findings of DM patients revealed an elevation in muscle enzyme levels, while the electromyogram indicated damage to the muscles. Approximately 1 to 6 out of every 100,000 adults in the United States are believed to suffer from DM (1). DM has a considerable genetic component, and some human leukocyte antigen (HLA) alleles are related to DM (2). For example, HLA-B∗08:01 has a significant association with adult-onset DM, and HLA-DRB1∗03:01 is associated with juvenile-onset DM (3). Additionally, the interferon (IFN) pathway has been demonstrated to play a role in DM (4), and cutaneous activity in adult DM is connected with a type I IFN gene signature (4).

Psoriasis is an immune-mediated chronic, recurrent, inflammatory, and systemic disease. Common clinical signs include localized or widely distributed scaly erythema or plaques. Psoriasis is caused by a combination of genetic, immune, and environmental factors (5). Genetic factors are the main risk factors for psoriasis development (6). HLA-C*06:02 is associated with the earlier-onset age of psoriasis (7). HLA-B27 may contribute to the susceptibility of psoriatic arthritis(PsA) (8), And the T helper (Th)17/interleukin (IL)-23 pathway is considered to be the primary pathway in psoriasis (9). Even HLA alleles play an important role in both diseases, there seems to be no report indicating that these two diseases share similar HLA haplotypes.

From a clinical perspective, psoriasis can occur alongside autoimmune conditions like autoimmune bullous diseases, vitiligo, alopecia, and thyroiditis (10), the concurrence of DM and psoriasis. Herein, we reported a case of psoriasis combined with DM, and a literature review was performed to speculate on the possible pathogenesis and treatment.

## 2 Case description

**Figure 1:**
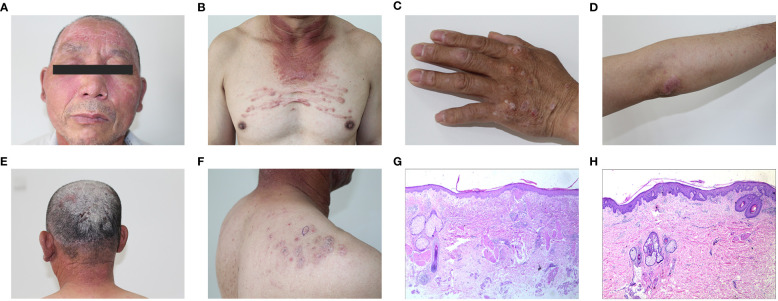
**(A)** Edematous violaceous erythema on the face and periorbital tissues. **(B)** Erythema on the neck and upper chest. **(C)** Left hand displaying Gottron’s papules. **(D)** The upper limb displays Gottron’s sign. **(E)** Scaly plaques on the scalp. **(F)** Scaly plaques on the back. **(G)** Histology from the facial lesion revealed liquefaction of basal cells, edema of the superficial dermis, infiltration of lymphocytes around small vessels with nuclear dust. **(H)** Skin biopsy from a scaly erythema lesion on the shoulder showed hyperkeratosis, parakeratosis, neutrophil accumulation, disappearance or thinning of the granular layer, mild thickening of the spinous layer, infiltration of lymphocytes and histiocytes around small vessels in the superficial dermis and deposition of mucinous substances in the interstitium.

In 2021, a 63-year-old Chinese male presented with a four-month history of infiltrative erythema on his face, neck, and upper chest, accompanied by muscle soreness and weakness in his limbs. Additionally, he had a 10-year history of psoriasis that only had topical therapies, and the symptoms were often recurrent. The physical examination revealed facial and periorbital edematous violaceous erythema (**Figure 1A**), erythema on the neck and upper chest (**Figure 1B**), Gottron’s papules (**Figure 1C**), Gottron’s sign (**Figure 1D**), scaly plaques on his scalp (**Figure 1E**) and back (**Figure 1F**). There was no arthralgia or nail involvement. Both the upper and lower limbs exhibited a grade 4 muscle strength accompanied by muscle tenderness.

Laboratory assay results revealed increased levels of lactate dehydrogenase (LDH) [417 U/L, normal range (NR) 120–246], aspartate aminotransferase (AST) (55 U/L, NR 15–46) and creatine kinase (CK) (229 U/L, NR 55–170). Biomarkers for lung cancer such as cytokeratin-19 fragment (CYFRA21-1) (27.1 ng/ml, NR 0–16.3) and neuron-specific enolase (NSE) (3.89 ng/ml, NR 0–3.3) were increased. However, his myositis-specific antibodies (MSAs) and autoantibody profiles were negative. Myogenic injuries to the right deltoid muscle and the biceps brachii muscle were seen on the electromyogram (EMG). A CT scan revealed chronic inflammation in the middle lobe on the right side and upper lobe on the left side of the lung, without any additional irregularities. Inflammation was detected in the lateral muscle groups of both shins through muscle MRI, along with the presence of effusion. A skin biopsy from the facial lesion revealed liquefaction of basal cells, edema of the superficial dermis, infiltration of lymphocytes around small vessels with nuclear dust (**Figure 1G**). Another skin biopsy from a scaly erythema lesion on his shoulder showed hyperkeratosis, parakeratosis, neutrophil accumulation, disappearance or thinning of the granular layer, mild thickening of the spinous layer, infiltration of lymphocytes and histiocytes around small vessels in the superficial dermis and deposition of mucinous substances in the interstitium (**Figure 1H**). The patient was diagnosed with dermatomyositis according to the Bohan and Peter’s criteria (11) and received treatment consisting of 24 mg/day methylprednisolone (MPSL), 400 mg/day hydroxychloroquine (HCQ), and 15 mg/week methotrexate(MTX). One month later, there was a notable relief in symptoms, with CK, LDH, and AST levels falling within the normal range. Additionally, the dosages of MTX and HCQ were reduced to 10 mg per week and 200 mg per day respectively. Subsequently, the doses of the three medications were gradually tapered, and now he receives 8 mg/day MPSL, 200 mg/day HCQ, and 5 mg/week MTX for treatment without any signs of recurrence. During the follow-up period, no clinical or radiological evidence of malignancy was observed in our patient.

## 3 Discussion

Autoimmune diseases like systemic lupus erythematosus (SLE), systemic sclerosis, and rheumatoid arthritis may coexist with psoriasis (10). Cases of psoriasis concurrent with dermatomyositis are also occasionally seen in daily clinical practice. As far as we know, there have been limited instances documented in the literature (**Table 1**) (12–23). In these cases, five were male and ten were female, of which six patients were under thirty years old, and they were all diagnosed with juvenile dermatomyositis. Six cases developed psoriasis prior to dermatomyositis, and dermatomyositis preceded in other cases. Among these cases, one had diabetes, hypertension, and anti-glomerular basement membrane disease, another had a hepatic tumor, a third had Hashimoto’s thyroiditis and Sjögren syndrome, and a fourth had interstitial lung disease.

**Table 1:** A summary of reported cases of DM coexistence with psoriasis.

	Age	Sex	Previous Situation	Interval Period	DM Subset	Psoriasis Subset	MSAs	Possible Trigger	Treatment
Pavlović MD et al. (12)	63	F	DM	4y	DM	psoriasis	N/A	N/A	MPSL, AZA
Gran JT et al. (13)	50s	M	psoriasis	N/A	DM	erythrodermic psoriasis	–	hepatic tumor	MPSL
Machado NP et al. (14)	51	M	DM	4y	DM	psoriasis	–	N/A	MPSL, CTX
Kim NN et al. (15)	18	F	JDM	9y	JDM	psoriasis	N/A	N/A	MTX
	8	F	psoriasis	10m	JDM	psoriasis	N/A	N/A	MPSL, MTX, MMF
	4	F	JDM	2y	JDM	psoriasis	N/A	N/A	TCS
Dicaro D et al. (16)	37	F	psoriasis	7y	CADM	psoriasis	–	adalimumab	MTX
Akiyama M et al. (17)	52	F	DM	8y	DM	psoriasis	anti-TIF-1 Ab	withdrawal of PSL	PSL, MTX
Montoya CL et al. (18)	20	M	CADM	5y	AJDM	erythrodermic psoriasis	–	N/A	ustekinumab
Inkeles MS et al. (19)	45	F	psoriasis	4y	CADM	psoriasis	N/A	N/A	CsA
Kato Y et al. (20)	30	F	DM	5y	DM	psoriasis	–	N/A	PSL
Xing Y et al. (21)	21	M	JDM	6y	JDM	Psoriasis	–	N/A	PSL, CsA, MTX
Schreiber C et al. (22)	45	F	PsA	N/A	DM	PsA	anti-Jo Ab	N/A	PED, MTX
Perna DL et al. (23)	20s	F	N/A	N/A	JDM	psoriasis	N/A	secukinumab	MTX
Present Case	63	M	psoriasis	10y	DM	psoriasis	–	sunlight exposure	HCQ, MPSL, MTX

F, female; M, male; DM, dermatomyositis; JDM, juvenile dermatomyositis; CADM, clinically amyopathic dermatomyositis; PsA, psoriatic arthritis; y, years; m, months; AJDM, amyopathic juvenile dermatomyositis; MSA, myositis-specific antibody; anti-TIF-1, anti-transcription intermediary factor 1 antibody; anti-Jo, Anti-Jo antibody; MPSL, methylprednisolone; AZA, azathioprine; CTX, cyclophosphamide; MTX, methotrexate; MMF, mycophenolate mofetil; TCS, topical corticosteroid; PSL, prednisolone; CsA, cyclosporin; PED, prednisone; HCQ, hydroxychloroquine.

Regarding the possible triggers, three cases might be associated with medications, including adalimumab, secukinumab, and withdrawal of prednisolone. One case might be linked to a hepatic tumor. As for our case, he had no medication history or underlying disease. He was a farmer who had been exposed to prolonged sunlight without any protection in this summer. We suspected that sunlight exposure might be the possible trigger.

Mechanistically, both DM and psoriasis are autoimmune diseases. Previous evidence has shown that these two diseases share some signaling pathways and cytokines (24, 25). For example, IFN can induce apoptosis and cause vascular damage directly (25), while TNF-α may also play a direct role in causing muscle inflammation in DM patients (25). In psoriasis, IFN can activate myeloid dendritic cells to secrete IL-12 and IL-23, and induce the activation and proliferation of Th1, Th17, and Th22 cells, resulting in the secretion of cytokines such as TNF-α, IL-17, and IL-22. These cytokines further stimulate keratinocytes, which produce related cytokines and chemokines to form inflammatory circuits and promote the characteristic changes in psoriasis (5). However, although psoriasis and dermatomyositis share some signaling pathways and cytokines, the mechanisms of their co-occurrence are still unclear. Based on previous studies, we speculate that there seems to be a complex, interacting, and self-sustaining inflammatory circuit among these cytokines in psoriasis (5). However, drugs such as adalimumab and secukinumab can disrupt the balance of the inflammatory circuit, leading to the accumulation or dominance of certain cytokines within inflammatory circuit and ultimately resulting in the appearance of clinical symptoms of DM, even though this condition is relatively rare (16, 23). As mentioned above, sunlight exposure may be a possible trigger for our patient. On one hand, ultraviolet (UV) radiation in sunlight is a proposed trigger for DM (26). On the other hand, UV radiation could suppress the IL-23/IL-17 axis, resulting in the inhibition of the production of IL-17 (27). This immune response results in a reduction of IL-17-mediated inflammation in skin lesions, which is similar to the effect of IL-17 inhibitors, and can also disrupt the inflammatory circuit. Accordingly, we think that UV radiation present in sunlight might play a significant role in the pathogenesis of the co-existence of dermatomyositis and psoriasis. However, further investigation is needed to confirm this point.

As for the treatment, 9 patients received corticosteroid treatment, 8 patients received MTX, other treatments included cyclosporin (CsA), cyclophosphamide (CTX), azathioprine (AZA), mycophenolate mofetil (MMF), HCQ, ustekinumab and topical corticosteroid (TCS). While two diseases share some inflammatory pathways and some therapy options could apply simultaneously, it could also be seen that treatment of one disease may exacerbate another one. For instance, UV phototherapy is safe and effective for psoriasis (28), but it is also a trigger for DM and even exacerbates the symptoms of DM (26). TNF-α inhibitors such as adalimumab, IL-17 inhibitors such as secukinumab, are effective for psoriasis, and are frequently used worldwide. However, there are studies reporting dermatomyositis or psoriasis occurring or exacerbating after receiving these two therapies (16, 23). And while TNF-α inhibitors may be a potential therapy for DM, the worsening of the disease can also be seen during treatment (29). Further observation is necessary when using TNF-α inhibitors to treat either both diseases simultaneously or only DM. Furthermore, corticosteroids are considered the preferred first-line therapy for DM-associated myopathy (30), but their use in psoriasis is not advised unless the situation is highly critical and the symptoms cannot be controlled by other therapies. Thus, when treating dermatomyositis accompanied by psoriasis, corticosteroids need to be used carefully. Clinically, immunosuppressive agents such as MTX are commonly given with corticosteroids to reduce the doses and side effects of corticosteroids (30). It seems to be most commonly combined with corticosteroid in treating dermatomyositis accompanied by psoriasis. Intravenous immunoglobulin (IVIG) was not mentioned in the treatment of the co-existence of dermatomyositis and psoriasis. On one hand, although IVIG shows efficacy in treating dermatomyositis (31), the evidence for the treatment of psoriasis is limited and may even lead to the aggravation of psoriasis (32). On the other hand, economic burden is a common reason for patients not to receive IVIG treatment. Additional drugs such as CsA (19), CTX (14) and ustekinumab (18) have shown efficacy in treating the concurrent presence of dermatomyositis and psoriasis. But these treatments are case reports, lacking high-quality evidence, and should only be for reference. Currently, the management of the simultaneous occurrence of dermatomyositis and psoriasis is still in the preliminary stage of investigation.

Limitations associated with this case report warrant mention. For example, the muscle biopsy and examination of the specific HLA alleles were not performed as consent was not obtained. Moreover, there is a lack of results for nail-fold capillary capillaroscopy, which is characteristic in dermatomyositis and could reflect the ongoing disease activity (33).

While dermatomyositis coexistence with psoriasis has been reported in various instances globally, this case stands out as the initial one to have achieved successful treatment through the combination of corticosteroids, methotrexate, and hydroxychloroquine. Herein, we report this case to provide some experience for clinical practice.

## Data availability statement

The original contributions presented in the study are included in the article/supplementary material. Further inquiries can be directed to the corresponding author.

## Ethics statement

Written informed consent was obtained from the individual(s), and minor(s)’ legal guardian/next of kin, for the publication of any potentially identifiable images or data included in this article.

## Author contributions

DC: Conceptualization, Writing – original draft. WY: Methodology, Writing – original draft, Writing – review & editing. JN: Investigation, Writing – review & editing.
